# Tumor-Targeted ZW800-1 Analog for Enhanced Tumor Imaging and Photothermal Therapy

**DOI:** 10.3390/pharmaceutics13101648

**Published:** 2021-10-09

**Authors:** Min-Ho Park, Gayoung Jo, Eun-Jeong Kim, Hoon Hyun

**Affiliations:** 1 Department of Surgery, Chonnam National University Medical School and Hwasun Hospital, Hwasun 58128, Korea; mhpark@jnu.ac.kr (M.-H.P.); angeleunei@naver.com (E.-J.K.); 2 Department of Biomedical Sciences, Chonnam National University Medical School, Hwasun 58128, Korea; jky6213@naver.com; 3 BioMedical Sciences Graduate Program (BMSGP), Chonnam National University, Hwasun 58128, Korea

**Keywords:** photothermal therapy, near-infrared fluorescence imaging, tumor targeting, zwitterionic fluorophores, ZW800-1

## Abstract

ZW800-1, a representative zwitterionic near-infrared (NIR) fluorophore, can minimize background tissue uptake owing to its balanced surface charges, and therefore, is widely used for improved NIR fluorescence imaging. As ZW800-1 has no tumor targetability, tumor imaging is highly dependent on the ability of the molecules conjugated to the ZW800-1. To enable tumor targeting using ZW800-1 without additional conjugation, we developed a tumor-targetable and renal-clearable ZW800-1 analog (ZW800-AM) based on the structural modification of ZW800-1. Specifically, an amine group on the center linker of the ZW800-1 indocyanine backbone was modified by replacing phenoxypropionic acid with tyramine linkage on the *meso*-chlorine atom. This modification improved the tumor targeting ability, which is known as the structure-inherent targeting strategy. More importantly, ZW800-AM not only showed sufficient tumor accumulation without nonspecific uptake but also produced a photothermal effect, killing tumor cells under 808 nm NIR laser irradiation. In addition, ZW800-AM exhibited rapid renal elimination from the body within 4 h of injection, similar to ZW800-1. Overall, the discovery of ZW800-AM as a bifunctional phototherapeutic agent may provide an ideal alternative for tumor-targeted imaging and phototherapy.

## 1. Introduction

The development of functional near-infrared (NIR) fluorophores with both target-specific imaging and cancer therapy capabilities has become of paramount importance for practical applications in NIR light-mediated photothermal cancer treatment [1,2,3]. Photothermal therapy (PTT), which is based on the principle of light-to-heat conversion, is a local and noninvasive cancer treatment approach by hyperthermia after administration of photosensitizers, leading to cancer cell death [4]. Among cyanine-based photosensitizers, the most well-known and the only clinically approved NIR fluorophore is indocyanine green (ICG) [5,6]. However, ICG has limitations in target-specific in vivo imaging owing to its poor bioavailability (e.g., solubility and stability), high liver uptake, and lack of functional groups for chemical conjugation with targeting molecules [7,8,9]. Previously, Choi et al. developed a zwitterionic NIR fluorophore, ZW800-1, with a balanced net surface charge and good water solubility, resulting in minimal background tissue uptake and rapid renal excretion [9,10,11]. In addition, the carboxylated form of ZW800-1 can further conjugate with various molecules for target-specific imaging [12,13,14,15,16,17,18,19]. Despite the significant improvements in ZW800-1 properties compared to those of other conjugatable NIR fluorophores (e.g., IRDye800CW and Cy5.5), the fundamental problem of low targeting efficiency is still unsolved because the targetability of ligands could be altered after conjugation [19].

Alternatively, several types of heptamethine cyanine fluorophores, such as IR-780, IR-783, and IR-808 (also called MHI-148), based on a structure-inherent targeting strategy without additional conjugation with targeting ligands have been extensively used for tumor-targeted imaging [20,21,22]. However, because these NIR fluorophores are relatively hydrophobic (log*D* values of 6.55, 3.63, and 4.48 at pH 7.4, respectively) compared with ZW800-1 (−3.35), they present cytotoxic effects originating from their nonspecific tissue/organ uptake and delayed excretion in the body [23]. To overcome these limitations, the development of optimal NIR fluorophores with improved physicochemical properties—tumor-targeting specificity, high water solubility, excellent optical properties, good biocompatibility, and rapid renal elimination from the body—is highly required.

Recently, we reported a tumor-targetable zwitterionic NIR fluorophore ZW800-Cl, as an intermediate of the ZW800-1 structure, used for tumor imaging and photothermal cancer therapy [24]. Although the *meso*-chlorine atom in the cyclohexenyl ring of ZW800-Cl played a key role in the formation of covalent albumin adducts, which are trapped in a tumor, ZW800-Cl showed nonspecific uptake in major organs such as lungs, liver, and spleen following 4 h of injection. In this study, we developed a tumor-targetable and renal-clearable zwitterionic NIR fluorophore, ZW800-AM, as an aminated ZW800-1 analog, in which the original center linker of the ZW800-1 skeleton is substituted with an amine-appended linker, improving tumor targetability to enable further application in effective PTT. ZW800-AM not only achieved preferential tumor accumulation without nonspecific tissue/organ uptake but also produced an excellent photothermal effect under NIR laser irradiation. To the best of our knowledge, this is the first report on a zwitterionic NIR fluorophore (ZW800-AM) that can realize both tumor-targeted imaging and effective PTT.

## 2. Materials and Methods

### 2.1. Synthesis of Zwitterionic NIR Fluorophores

Solvents and reagents were purchased from Sigma-Aldrich (St. Louis, MO, USA) as reagent grade and used without further purification. The ZW800-1 and ZW800-AM heptamethine cyanine fluorophores were prepared as described previously [9,10,25]. ZW800-AM was synthesized as follows: a mixture of sulfonated indolium salt (0.15 g, 0.28 mmol), Vilsmeier−Haack reagent (0.05 g, 0.14 mmol), and anhydrous sodium acetate (0.04 g, 0.42 mmol) in anhydrous ethanol (5 mL) was refluxed for 6 h. The reaction mixture was cooled to ambient temperature and then filtered, washed with ethanol and methanol, and obtained as a dark green solid (ZW800-Cl; 0.1 g, 90%). Before introducing a tyramine linkage on the *meso*-chlorine atom, tert-butyloxycarbonyl (Boc)-protected tyramine was prepared by adding triethylamine (0.23 g, 2.28 mmol) and Boc anhydride (0.5 g, 2.29 mmol) into tyramine solution (0.21 g, 1.53 mmol) in dimethylformamide (DMF; 5 mL). The reaction mixture was stirred at ambient temperature for 2 h. To the above solution, under nitrogen atmosphere, sodium hydride (0.04 g, 1.6 mmol) was added, and the mixture was stirred at ambient temperature for 1 h. Subsequently, ZW800-Cl (0.1 g, 0.12 mmol) was added to the above solution and the mixture was stirred at room temperature for 17 h. For the Boc deprotection, a solution of trifluoroacetic acid (TFA) and water (5 mL, 50/50 *v*/*v*%) was mixed with the above solution and stirred at room temperature for additional 2 h. The crude mixture was crystallized with ethyl acetate, collected, and dried in a vacuum chamber. The final product was purified using a preparative high-performance liquid chromatography (HPLC) system (Waters, Milford, MA, USA). The molecular weight of the ZW800-AM was identified using an ultra-performance liquid chromatography (UPLC, Waters) system combined with micrOTOF-Q II (Bruker, Ettlingen, Germany).

### 2.2. Optical and Physicochemical Property Analyses

All optical measurements were performed in phosphate-buffered saline (PBS) at pH 7.4. The absorption spectra of ZW800-1 and ZW800-AM were recorded using a fiber optic UV-Vis-NIR (200–1025 nm) spectrophotometer (Ocean Optics, Dunedin, FL, USA). The molar extinction coefficient (*ε*) was determined based on the Beer−Lambert equation. ICG (*Φ* = 13% in DMSO) was used as a calibration standard to measure the fluorescence quantum yields of ZW800-1 and ZW800-AM under the conditions of matched absorbance at 770 nm [9,10,25]. The fluorescence spectra of ZW800-1 and ZW800-AM were measured by a SPARK^®^ 10M microplate reader (Tecan, Männedorf, Switzerland) at the 700 nm excitation in the wavelength range of 750–900 nm. In silico predictions of the partition coefficient (log*D* at pH 7.4) and the topological polar surface area (TPSA) for ZW800-1 and ZW800-AM were calculated using Marvin and JChem calculator plugins (ChemAxon, Budapest, Hungary).

### 2.3. In Vitro Cell Binding and NIR Fluorescence Microscopy

Human large-cell lung carcinoma cell line NCI-H460 and mouse embryonic fibroblast cell line NIH/3T3 were obtained from the American Type Culture Collection (ATCC, Manassas, VA, USA). Cells were cultured in Roswell Park Memorial Institute (RPMI) 1640 or Dulbecco’s Modified Eagle Medium (DMEM) media (Gibco BRL, Paisley, UK) supplemented with 10% fetal bovine serum (Gibco BRL) and an antibiotic–antimycotic solution (Welgene, Daegu, Korea) and placed in a 5% CO_2_ incubator at 37 °C. When a cell line reached about 50% confluence, the cells were washed with PBS. Subsequently, ZW800-AM was added to each well in the range of 2–20 μM concentrations. The cells were placed in an incubator at 37 °C for 24 h and then washed with PBS. NIR fluorescence imaging was performed using a Nikon Eclipse Ti-U inverted microscope system (Nikon, Seoul, Korea).

### 2.4. In Vitro Cytotoxicity Assay

Cell toxicity and proliferation were evaluated using an alamarBlue^TM^ (Thermo Scientific, Waltham, MA, USA) assay. NCI-H460 cells were seeded onto 96-well plates (1 × 10^4^ cells per well). To determine the cytotoxicity as a function of the concentration, the cancer cells were treated with ZW800-AM (2, 10, 25, and 50 μM) for 1 h and cultured for 24 h after treatment. At each assay time point, the incubation cell medium was replaced with 100 μL of fresh medium, and 10 μL of the alamarBlue solution was directly added to each 100 μL well. Subsequently, the plates were placed in a 5% CO_2_ incubator at 37 °C for 4 h. Finally, the plates were analyzed using a microplate reader (SPARK^®^ 10M, Tecan) to determine the intensities of the absorbance at 570 nm and the fluorescence emission at 590 nm. Cell viability was determined based on the following formula: cell viability (%) = (*A*_sample_ − *A*_blank_)/(*A*_control_ − *A*_blank_) × 100, where *A* is the average absorbance.

### 2.5. NCI-H460 Xenograft Mouse Model

Animal protocols were in accordance with the guidelines of the Chonnam National University Animal Research Committee (CNU IACUC-H-2020-19). Male NCRNU mice (6 weeks old, ≈25 g) were purchased from OrientBio (Seongnam, Korea). Cultured NCI-H460 cells were suspended in PBS before they were subcutaneously injected in the right flank of each mouse (1 × 10^6^ cells per mouse). When tumor sizes reached approximately 1 cm in diameter, ZW800-AM or ZW800-1 was administered intravenously. The animals were euthanized and imaged over a certain period.

### 2.6. In Vivo Biodistribution and Tumor Imaging

Time-dependent NIR fluorescence imaging was conducted using an in vivo NIR fluorescence imaging system (FOBI, NeoScience, Suwon, Korea). Mice were sacrificed at 4 h post-injection of ZW800-AM, and their organs were collected and imaged to confirm the biodistribution of ZW800-AM. The fluorescence intensities in the tumors and resected organs were measured using the open sourced ImageJ software (National Institutes of Health, Bethesda, MD, USA).

### 2.7. In Vivo Photothermal Therapeutic Efficacy

ZW800-AM or PBS were administered intravenously to the NCI-H460 tumor-bearing mice and the mice were anaesthetized 2 h after injection. The tumors were exposed to 808 nm laser irradiation (1.1 W/cm^2^) for 5 min. Tumor temperature was monitored through a thermal imager (FLIR Systems, Wilsonville, OR, USA). Subsequently, tumors were harvested from each group 24 h post-irradiation for histological assessment after staining with hematoxylin and eosin (H&E). The macroscopic tumor growth of each group was observed to verify the photothermal therapeutic efficacy for 9 days. The tumor volume (V) was measured using the following formula: V = 0.5 × longest diameter × (shortest diameter)^2^.

### 2.8. Statistical Analysis

Statistical analysis was conducted by one-way analysis of variance for multiple comparison test. The results were expressed as mean ± standard deviation (S.D.). A value of *p* < 0.05 was used as the statistically significant.

### 2.9. Histological Analysis

Tumors excised from each group were stored for H&E staining and microscopic analysis. The tumor sections were fixed in 4% paraformaldehyde and placed in a deep freezer. Frozen tumors were cryosectioned (10 µm thick) and stained with H&E. Histological analysis was conducted on a Nikon Eclipse Ti-U inverted microscope system (Nikon).

## 3. Results and Discussion

### 3.1. Synthesis and Characterization of ZW800-AM

**Figure 1:**
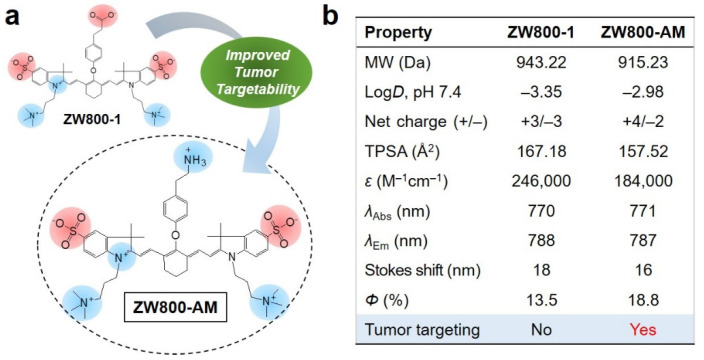
(**a**) Chemical structures of ZW800-1 and ZW800-AM NIR fluorophores, and (**b**) their physicochemical and optical properties in PBS at pH 7.4. In silico calculations of log*D* at pH 7.4 and TPSA are conducted using Marvin and JChem calculator plugins (ChemAxon, Budapest, Hungary).

The balanced surface charge of the ZW800-1 structure is the most important characteristic for its excellent in vivo performance, including minimal background tissue uptake and rapid renal clearance because of the low plasma protein binding [9,10,11]. Owing to the lack of its tumor specificity, the carboxylated form of ZW800-1 has been widely used for in vivo NIR fluorescence imaging of cancer after conjugation with tumor-targeting ligands such as cyclic RGD peptide and sorbitol [12,14]. Herein, we report for the first time the tumor-targeting ability of ZW800-AM, which is an aminated form of ZW800-1, without additional conjugation with tumor-targeting ligands. As depicted in Figure 1a, ZW800-1 and ZW800-AM have similar chemical structures except for the functional groups on the center linker that maintains the balanced surface charge, which plays a critical role in tumor targetability. Moreover, in silico calculations of the physicochemical properties—hydrophobicity (log*D*), net surface charge, and polarity (TPSA)—provided comparative information about ZW800-AM (Figure 1b). Importantly, the lower hydrophilicity and polarity of ZW800-AM compared to those of ZW800-1 as well as its net positive charge may contribute to a significant difference in terms of tumor targetability.

**Figure 2:**
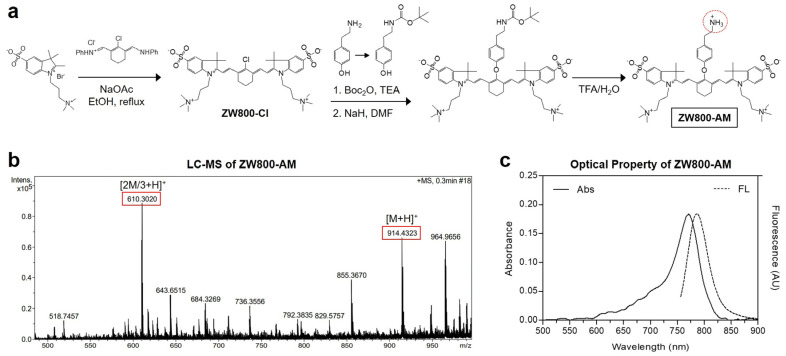
(**a**) Synthesis scheme, (**b**) mass spectrum, and (**c**) absorbance and fluorescence emission spectra of ZW800-AM NIR fluorophore. Optical measurements are performed in PBS at pH 7.4.

As shown in Figure 2a, ZW800-AM is synthesized by the conjugation of the chloro-substituted ZW800-Cl with tyramine. The chloro-substituted heptamethine cyanine fluorophore, ZW800-Cl, was prepared via a condensation reaction between the Vilsmeier−Haack reagent and zwitterionic indolium salts, using the procedure developed by Choi et al. [9,10,25]. Subsequently, the phenoxide ion of Boc-protected tyramine was conjugated with the *meso*-chlorine atom in the cyclohexenyl ring of ZW800-Cl via a nucleophilic displacement reaction. To increase the nucleophilicity of Boc-protected tyramine, sodium hydride was used to generate phenoxide ions in situ. In the final step, the Boc protection was removed by carbamate hydrolysis in acidic conditions to form the primary amine group of ZW800-AM. The final product was separated using a preparative HPLC system and analyzed via liquid chromatography–mass spectrometry (LC-MS) to determine the molecular weight of ZW800-AM (Figure 2b). As ZW800-AM is basically derived from the ZW800-1 structure, its optical properties were similar to those of ZW800-1. The peak absorption and fluorescence emission spectra of ZW800-AM in the NIR region were obtained at 771 and 787 nm, respectively (Figure 2c). This suggests that ZW800-AM is suitable for use in NIR laser-induced PTT.

### 3.2. In Vitro Cancer Cell Binding and Cytotoxicity

**Figure 3:**
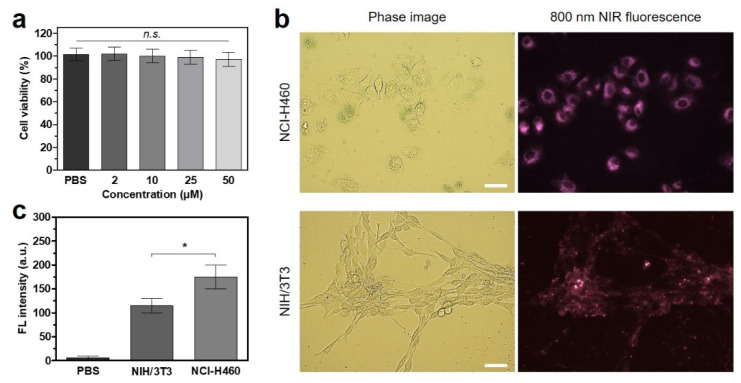
(**a**) Cytocompatibility assay of ZW800-AM using the NCI-H460 cell line. The percentage of cytotoxicity versus concentration is determined at 24 h post-treatment of ZW800-AM in the range of 2–50 μM concentrations. (**b**) Live cell imaging of ZW800-AM in NCI-H460 and NIH/3T3 cells. Phase and NIR fluorescence images of each cell line are representative (*n* = 3) and monitored at 24 h post-treatment with 20 µM ZW800-AM. All NIR fluorescence images have identical exposure times and normalization. Scale bar = 100 μm. (**c**) Relative fluorescence intensities in NCI-H460 and NIH/3T3 cells at 24 h post-treatment with 20 µM ZW800-AM. Data are expressed as mean ± S.D. (*n* = 3). * *p* < 0.05; *n.s.* = not significant.

The cytotoxicity and cellular binding of ZW800-AM were investigated using the NCI-H460 cancer cell line. To determine the cell viability, the alamarBlue assay was conducted in NCI-H460 cells after incubation with ZW800-AM at various concentrations (2–50 μM). Interestingly, ZW800-AM showed no significant cytotoxicity to the NCI-H460 cancer cells even at high concentrations of 25–50 μM, demonstrating good biocompatibility (Figure 3a). Moreover, we observed the intracellular distribution of ZW800-AM via NIR fluorescence microscopy after 24 h of incubation in NCI-H460 and NIH/3T3 cells. As expected, ZW800-AM presented significant intracellular localization with high fluorescence signals in the cytoplasm due to its net positive charge (Figure 3b). In contrast with ZW800-1, which shows no cellular uptake under any conditions owing to its balanced surface charge [12], the positive surface charge of ZW800-AM may play an important role in binding it to the negatively charged outer surfaces of cell membranes. This suggests that ZW800-AM can bind to cancer cells, thereby having the potential to deliver thermal energy to cell membranes, leading to cancer cell death. Interestingly, the ZW800-AM exhibited relatively weak fluorescence intensity in normal cells compared with NCI-H460 cancer cells (Figure 3c). Although differences in the binding affinities of ZW800-AM between normal and cancer cells are still under investigation, there are more important factors not only its positive charges but also receptor-mediated binding more favorable to cancer cells.

### 3.3. Time-Dependent In Vivo Tumor Imaging and Biodistribution

**Figure 4:**
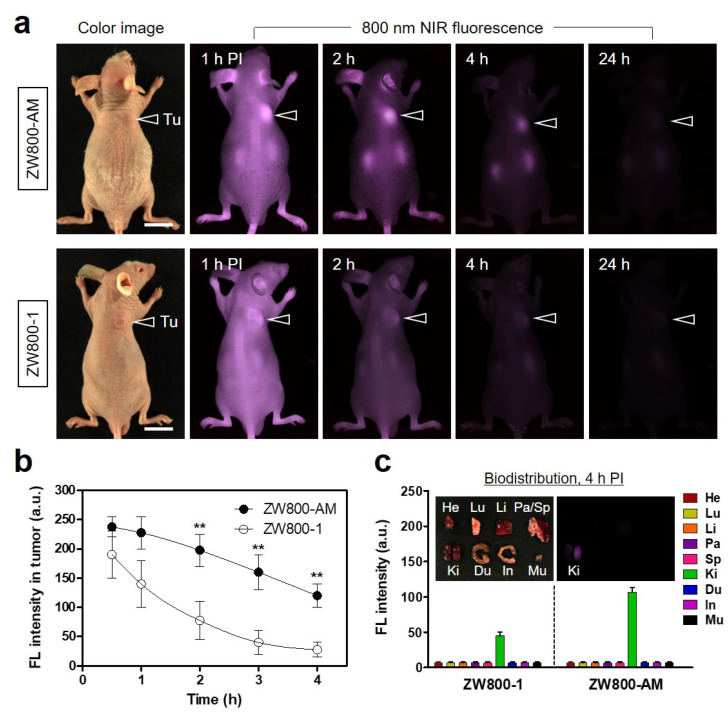
(**a**) Real-time NIR fluorescence imaging for 24 h post-injection of ZW800-AM and ZW800-1 (*n* = 3). Tumor sites are indicated by arrowheads. (**b**) Fluorescence intensities in tumors as a function of post-injection time of ZW800-AM and ZW800-1. Data are expressed as mean ± S.D. (*n* = 3). ** *p* < 0.01. (**c**) Quantitative fluorescence intensities of resected organs at 4 h post-injection of ZW800-AM and ZW800-1. Inset shows excised organs imaged at 4 h post-injection of ZW800-AM. Abbreviations: Du, duodenum; He, heart; In, intestines; Ki, kidneys; Li, liver; Lu, lungs; Mu, muscle; Pa, pancreas; Sp, spleen; Tu, tumor; PI, post-injection. Scale bars = 1 cm. All NIR fluorescence images have identical exposure times and normalization.

To investigate the in vivo tumor targetability of ZW800-AM, NCI-H460 tumor-bearing mice were intravenously injected with 10 nmol ZW800-AM or ZW800-1 (as the control group) and monitored for 24 h using a real-time NIR fluorescence imaging system (Figure 4a). Time-dependent NIR fluorescence imaging showed rapid tumor accumulation in the mice injected with ZW800-AM until 4 h after the injection; in contrast, no tumor specificity was observed in the control group treated with ZW800-1, which is consistent with the well-known characteristics of ZW800-1 reported previously [19,24]. A high fluorescence intensity observed at the tumor treated with ZW800-AM was maintained for approximately 2 h after the injection, whereas the fluorescence signal at the tumor treated with ZW800-1 continuously decreased without tumor-specific accumulation (Figure 4b). To the best of our knowledge, this is the first report on a zwitterionic NIR fluorophore, ZW800-AM, enabling tumor-targeted imaging for further application in photothermal cancer therapy. Considering the optimal time point for the subsequent PTT and based on the tumor-to-background signal ratio, the photothermal treatment can be performed 2 h, instead of 1 h, after the ZW800-AM injection to prevent unnecessary damage to neighboring normal tissues. Moreover, the biodistribution and clearance of ZW800-AM were investigated by comparing the fluorescence signals in major organs after 4 h of injection (Figure 4c). Importantly, ZW800-AM did not show any nonspecific tissue/organ uptake 4 h after injection owing to its rapid renal excretion, which is highly similar to the excellent in vivo performance of ZW800-1, as reported previously [9,10,11,25]. Although ZW800-AM possesses a net positive surface charge unlike the balanced net charge of ZW800-1, the former underwent rapid and complete urinary elimination within 4 h of injection. This result demonstrates that the amine group of the ZW800-AM structure only played a key role in the tumor targeting and may not affect the biodistribution during the circulation in blood. Therefore, ZW800-AM has significant potential to be used as a PTT agent capable of rapid tumor targeting and high renal clearance.

### 3.4. In Vitro and In Vivo Photothermal Effects

**Figure 5:**
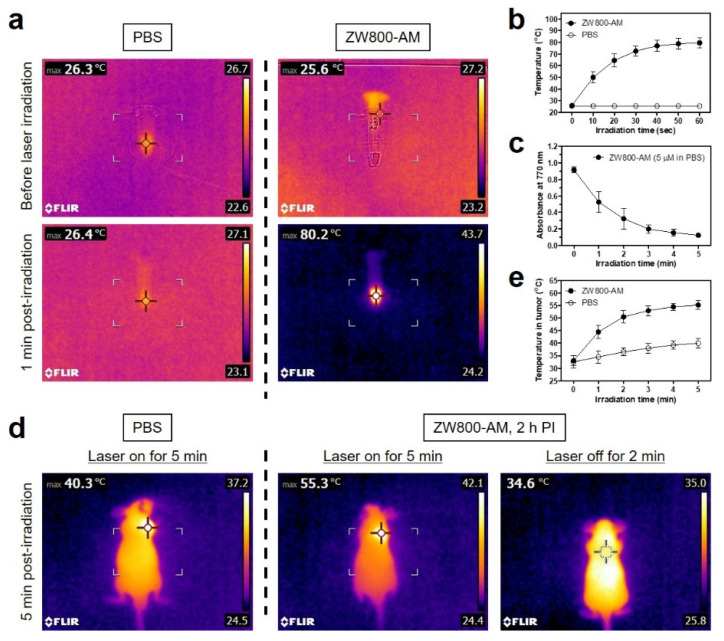
(**a**) In vitro thermal images of ZW800-AM solution (100 μM dissolved in PBS) and only PBS (100 μL) before and after 808 nm laser irradiation (1.1 W/cm^2^) for 60 s. The maximum temperature is automatically recorded with an infrared thermal camera. (**b**) Temperature changes in each solution are observed for 60 s of laser irradiation. (**c**) Photostability of ZW800-AM during 5 min of laser irradiation. Absorbance changes are measured at 770 nm using 5 μM ZW800-AM solutions under laser irradiation. (**d**) In vivo thermal images of tumor-bearing mice at 2 h post-injection of PBS or ZW800-AM after 808 nm laser irradiation (1.1 W/cm^2^) for 5 min. (**e**) Temperature changes of tumors in each group are observed for 5 min of laser irradiation. Data are expressed as mean ± S.D. (*n* = 3).

To confirm the photothermal properties of ZW800-AM, ZW800-AM (100 μM in PBS) and PBS solutions were individually exposed to 808 nm laser irradiation (1.1 W/cm^2^) for 1 min. Previously, the power density of an 808 nm laser was determined optimal, capable of avoiding the unnecessary photothermal effect of only laser power, without any PTT agent [26,27]. Temperature changes during the laser irradiation were automatically recorded using an FLIR^®^ thermal imager as a function of the irradiation time. Interestingly, the temperature of the ZW800-AM solution rapidly increased from 25.6 to 80.2 °C under the laser irradiation for 1 min, whereas the PBS solution alone showed no change in temperature under the same condition (Figure 5a). The photothermal temperature of ZW800-AM was remarkably raised to ~70 °C during the first 30 s of laser irradiation and maintained up to ~80 °C during the next 30 s of irradiation (Figure 5b). This suggests that ZW800-AM can be used as an efficient PTT agent for photothermal cancer treatment. Based on the light-to-heat conversion capability of ZW800-AM in vitro, its photothermal conversion efficiency (*η*) was calculated as 30.5% using a previous method [28], which is comparable to that of ZW800-1 conjugates (32.6–34.1%) reported previously [15,19]. Additionally, the absorbance changes of ZW800-AM were repeatedly measured at 770 nm during 5 min of laser irradiation to test its photostability against continuous photoexcitation. The absorbance of the ZW800-AM solution steadily decreased after 3 min of laser irradiation, indicating that its cyanine structure was finally destroyed by photobleaching on exposure to localized NIR light (Figure 5c).

The in vivo photothermal conversion capability of ZW800-AM was further investigated using NCI-H460 tumor-bearing mice. ZW800-AM and PBS solutions were intravenously injected into the mice 2 h before laser irradiation, and the tumor sites were subsequently exposed to 808 nm laser irradiation at 1.1 W/cm^2^ for 5 min. The tumor temperatures in the ZW800-AM-injected mice remarkably increased up to ~55 °C, whereas the PBS-treated mice exhibited little change in the tumor temperature (~40°C) after 5 min of laser irradiation (Figure 5d). Additionally, the higher tumor temperature was recovered to the body temperature (~34 °C) after 2 min of laser off. Particularly, the peak temperature in the tumors plateaued after 3 min of laser irradiation and were maintained up to ~55 °C until the next 2 min of irradiation, which is notably sufficient to induce complete necrosis in tumor tissues (Figure 5e). This result demonstrates that ZW800-AM can be used for targeted photothermal cancer therapy with excellent light-to-heat conversion efficiency.

### 3.5. In Vivo Photothermal Therapeutic Efficacy

**Figure 6:**
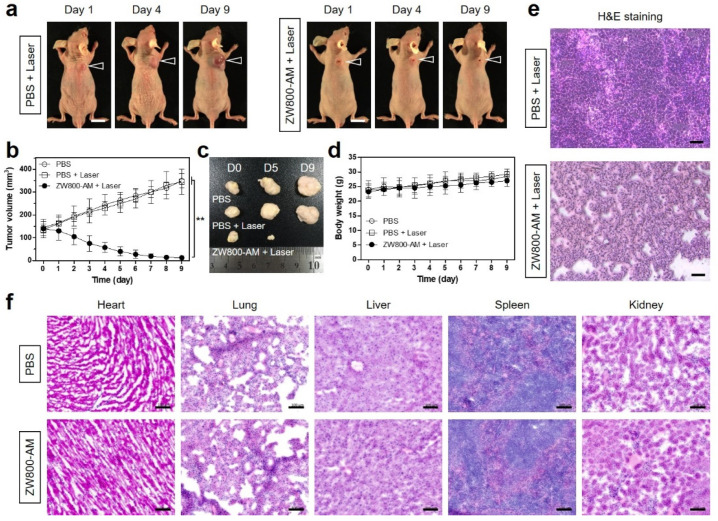
In vivo NIR phototherapeutic efficacy. (**a**) Representative photos of tumor size changes in NCI-H460 tumor-bearing mice over 9 days after different treatments. Laser groups are treated with 2 h post-injection of PBS and ZW800-AM, followed by 808 nm laser irradiation (1.1 W/cm^2^) for 5 min. Tumor sites are indicated by arrowheads. Scale bars = 1 cm. (**b**) Tumor growth rates, (**c**) gross tumor photos, and (**d**) body weights of each treatment group were monitored for 9 days. Data are expressed as mean ± S.D. (*n* = 3). ** *p* < 0.01. (**e**) Tumor sections stained with H&E from each group after 24 h of different treatments. (**f**) H&E stained images of major organs including heart, lung, liver, spleen, and kidney tissues after PBS and PTT treatments. Images are representative of three independent experiments. Scale bars = 100 μm.

The in vivo phototherapeutic efficacy of ZW800-AM was confirmed by monitoring tumor sizes for 9 days after the photothermal treatment (Figure 6a). Laser irradiation with only PBS treatment did not induce skin damage (burn scarring) and tumor suppression. Concurrently, laser irradiation of tumor-bearing mice injected with ZW800-AM showed a remarkable PTT effect with complete tumor ablation and no remarkable recurrence during the course of the treatment (Figure 6b,c). This result demonstrates that tumor growth is successfully inhibited by combining of ZW800-AM injection and NIR laser irradiation. More importantly, the therapeutic efficacy of PTT highly depends on the tumor location in the body, because of the low penetration depth of an NIR laser light. In terms of the orthotopic tumor models for PTT, the skin or liver cancers compared to other types of cancer could be applicable for photothermal cancer treatment in consideration of the limited light penetration depth.

In addition, the body weight of each group was monitored for 9 days, and the treatment groups exhibited normal variation during the therapeutic process, indicating that no significant side effects occurred (Figure 6d). Furthermore, the phototherapeutic effect was confirmed by the H&E staining of the tumor tissues harvested from each group 24 h after the different treatments (Figure 6e). The H&E staining of the tumor sections belonging to the PBS and laser-treated group showed no cell damage, with typical morphological features of cell proliferation. However, the tumor sections treated with ZW800-AM and laser irradiation revealed notable features of necrotic cell death with a reduced cell number and shrunken nuclei. This indicates that ZW800-AM can be used as an efficient PTT agent for generating thermal energy and consequently inducing cell apoptosis and necrosis. Moreover, histological assessment of the major organs (heart, lung, liver, spleen, and kidney) resected from each group showed no pathological changes or lesions (Figure 6f). This result confirms the biosafety of ZW800-AM without causing systemic toxicity owing to its rapid distribution and elimination characteristics.

## 4. Conclusions

In this study, we identified a zwitterionic NIR fluorophore, ZW800-AM as a ZW800-1 analog, which could be used for tumor-targeted imaging and PTT simultaneously without further conjugation with tumor-specific ligands and photosensitizers. Although several types of cyanine-based NIR fluorophores have been developed for tumor-targeted imaging with structure-inherent targeting capacity, the biosafety issues associated with their nonspecific tissue/organ uptake still remain unsolved. Most importantly, ZW800-AM showed rapid renal excretion within 4 h of injection without nonspecific uptake in the body, which could overcome the issues related to the longstanding biosafety problems for the clinical use of contrast agents. Currently, ZW800-1 is one of the most well-known fluorophores used for in vivo NIR fluorescence imaging because of its excellent in vivo performance; however, this is achieved after conjugation with various small molecules, proteins, or nanoparticles. In this regard, ZW800-AM may be an alternative candidate for cancer theranostics with tumor targetability as well as excellent optical properties and biocompatibility. Overall, the tumor-targetable, renal-clearable, and PTT-applicable ZW800-AM has a strong potential in future clinical applications.

## Data Availability

Not applicable.
